# A Small Molecule BH3-mimetic Suppresses Cigarette Smoke-Induced Mucous Expression in Airway Epithelial Cells

**DOI:** 10.1038/s41598-018-32114-w

**Published:** 2018-09-14

**Authors:** Shah S. Hussain, Shebin George, Shashi Singh, Rahul Jayant, Chien-An Hu, Mohan Sopori, Hitendra S. Chand

**Affiliations:** 1 Department of Immunology & Nano-Medicine, Institute of NeuroImmune Pharmacology, Herbert Wertheim College of Medicine, Florida International University, Miami, FL - 33199 USA; 2 Lovelace Respiratory Research Institute, Albuquerque, NM - 87108 USA; 3 Department of Biochemistry and Molecular Biology, University of New Mexico, Albuquerque, NM - 87131 USA

## Abstract

Cigarette smoke (CS) exposure is one of the primary risk factors associated with the chronic mucous hypersecretion (CMH). The antiapoptotic protein, Bcl-2 sustains hyperplastic mucous cells, and the airway epithelium of ex-smokers with CMH as well as mice exposed to chronic CS showed increased Bcl-2 expression. Therefore, we investigated whether Bcl-2 plays a role in CS-induced mucous expression. Primary airway epithelial cells (AECs) of murine and human origin were treated with CS extract (CSE), and there was a concentration- and time-dependent increase in secretory mucin (MUC5AC), mucous regulator (SPDEF) and Bcl-2 expression. Using differentiated human AECs cultured on air-liquid interface, EGFR and ERK1/2 pathways were interrogated. Bcl-2 activity was blocked using a small molecule BH3 mimetic ABT-263 that disrupts the Bcl-2 interaction with pro-apoptotic proteins. The ABT-263 treatment resulted in the downregulation of CSE-induced mucus expression and disrupted the EGFR-signaling while inducing the apoptosis and the pro-apoptotic protein, Bik expression. This strategy significantly suppressed the mainstream CS-induced mucous phenotype in a 3-D human airway epithelium model. Therefore, the present study suggests that CS induces Bcl-2 expression to help promote mucous cell survival; and small molecule BH3 mimetics targeting Bcl-2 could be useful in suppressing the CS-induced mucous response.

## Introduction

Airway mucus secretion plays a key role in innate immune responses against inhaled toxicants and pathogens. However, in susceptible population there is abnormally high level of mucus production and accumulation in the airways, specifically in patients suffering from chronic mucus hypersecretion (CMH)^1,2^. The primary mechanisms associated with CMH are mucus overproduction and hypersecretion by the goblet or mucous cells and the decreased elimination of mucus. CMH prevalence varies from 3.5% to 12.7% in the general population but is much higher (~30%) in individuals with COPD^1,3^. In CMH patients, the airway epithelial responses are compromised due to dysregulated mucus production, increased mucous cell numbers and ineffective airway clearance^1,4^. This mucous phenotype is highly exacerbated in patients affected with severe COPD and the poorly controlled CMH leads to airway plugging and reduced lung functions^5–10^. Therefore, understanding the molecular mechanisms responsible for the increased differentiation and proliferation of hyperplastic mucous cells and resulting mucus overexpression and hypersecretion are crucial in developing CMH targeted therapeutics.

Cigarette smoke (CS) exposure is one of the primary risk factors associated with CMH and the debilitating mucus hyperproduction^11,12^. CS exposure alters the cell fate by affecting the cell proliferation and the cell death pathways^13–17^. One of the plausible mechanism could involve modulating the levels of Bcl-2, an anti-apoptotic protein that promotes cell survival^13,18–20^. In support of this, we have shown that airway inflammation induces Bcl-2 in airway epithelium and induced Bcl-2 sustains the survival of hyperplastic mucous cells^14,15,20–22^. Furthermore, our recent findings showed that Bcl-2 is one of the main drivers associated with the airway mucous responses^14,15,20^, therefore, the effect of CS exposure on Bcl-2 expression was investigated in this study. The secretory mucin that is primarily produced by mucous cells in the airway epithelium is MUC5AC, which is induced upon CS exposure and other airway injuries^8,23,24^. In chronic airway diseases such as COPD and asthma, the debilitating mucus or phlegm production is highly associated with increased numbers of mucous cells with increased mucin synthesis and secretion^8^ and this pathology is primarily driven by MUC5AC, as shown by a recent study^25^.

In an animal model of chronic CS exposure, we had observed increased expression of Bcl-2 mRNA in mice exposed to CS for 16 weeks with 4-fold higher number of airway epithelial cells (AECs) showing Bcl-2 immunopositivity in CS-exposed mice compared to air-exposed controls^22^. More importantly, bronchial biopsies from ex-smokers with CMH showed significantly increased Bcl-2 levels with 5-fold increased immunopositivity compared to control subjects^20^. Therefore, we investigated the role of Bcl-2 in CS-induced mucous expression using cultured murine and human airway epithelial cells and tested whether targeting Bcl-2 using a small molecule BH3 mimetic compound, ABT-263, could help in modulating CS-induced mucous expression.

## Results

### CS induces mucus and Bcl-2 levels in a concentration- and time-dependent manner in murine AECs

**Figure 1:**
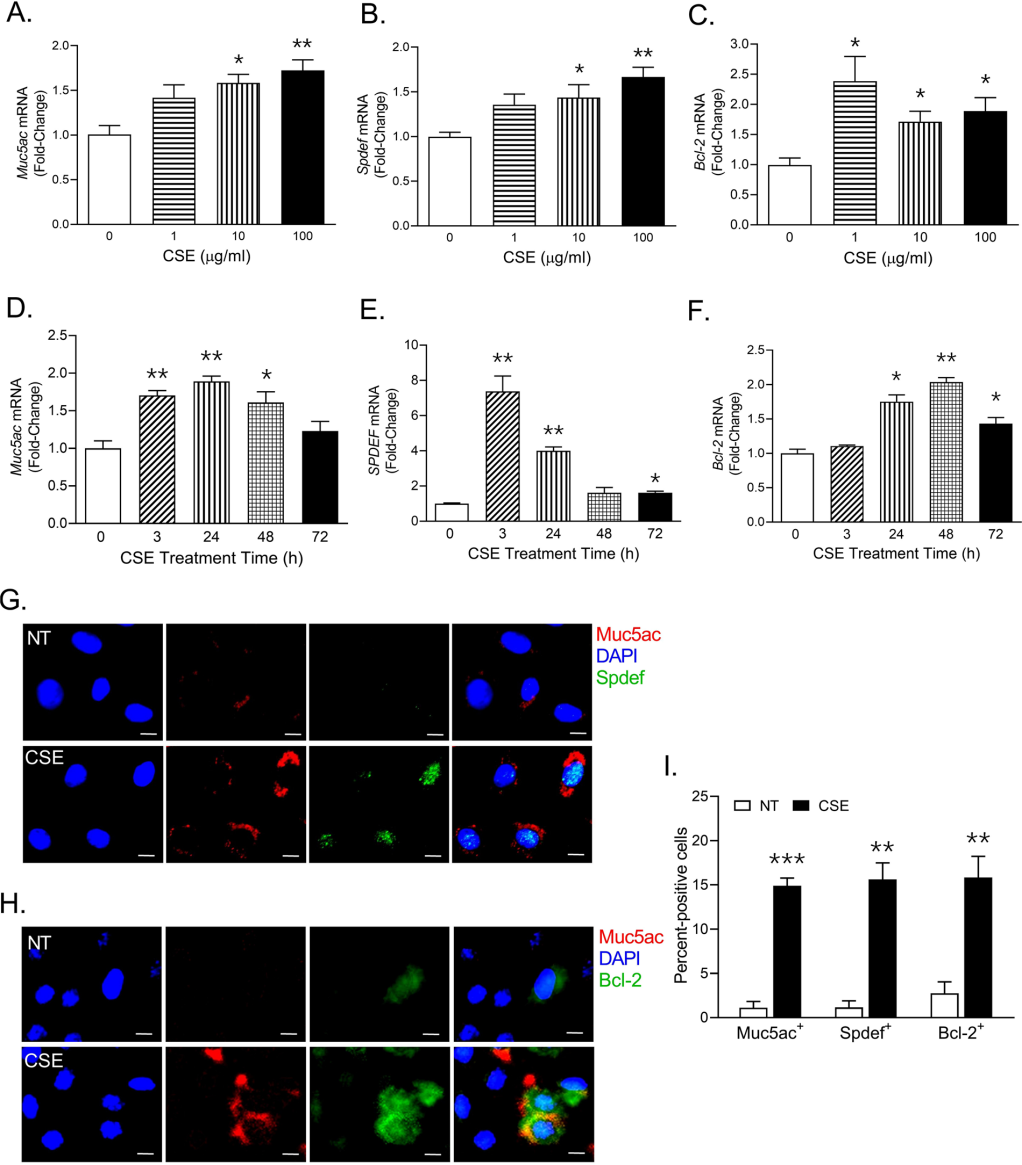
CS exposure induces mucous phenotype and Bcl-2 levels in murine airway epithelial cells (AECs). Primary murine AECs were treated with cigarette smoke extract (CSE) at 0, 1, 10, and 100 µg/ml for 24 h and the mRNA levels of *Muc5ac* (**A**) *Spdef* (**B**) and *Bcl-2* (**C**) were analyzed by qRT-PCR. Murine AECs treated with 10 µg/ml CSE and cells were harvested at 0, 3, 24, 48 and 72 h and mRNA levels of *Muc5ac* (**D**) *Spdef* (**E**) and *Bcl-2* (**F**) were quantified. (**G**) Representative micrographs showing Muc5ac (red) and Spdef (green) expression in murine AECs following CSE treatment in comparison with non-treated (NT) cells. Murine AECs were treated with CSE (10 µg/ml) for 48 h and immunostained for Muc5ac and Spdef, and the nuclei (blue) were stained with DAPI (Scale – 5 µ). (**H**) Representative micrographs showing Muc5ac (red) and Bcl-2 (green) expression in CSE-treated and treated (NT) murine AECs, nuclei were stained with DAPI (Scale – 5 µ). (**I**) Quantification of Muc5ac, Spdef and Bcl-2 immunopositive cells following CSE exposure. Approximately 300 cells from each treatment were analyzed to calculate the percentage of Muc5ac-positive (Muc5ac^+^), Spdef-positive (Spdef^+^) and Bcl-2-positive (Bcl-2^+^) cells. Data shown as mean ± SEM (n ≥ 3); *p < 0.05; **p < 0.01; ***p < 0.001.

CS induces mucus production and mucous cell hyperplasia in airway epithelium^13,16,26,27^, nonetheless, the molecular mechanisms involved in CS-induced mucous expression remain elusive. We analyzed the effect of CS extract (CSE) on primary murine AECs by treating them with 0, 1, 10 and 100 µg/ml of CSE for 24 h. Cells were analyzed for the expression of a secretory mucin, Muc5ac^8,28^; a master transcriptional regulator of mucous response, Spdef or SAM pointed domain containing ETS transcription factor^29^; and Bcl-2, a key anti-apoptotic protein that sustains mucous cells^14,15,20,21^. There was a dose-dependent increase in *Muc5ac* mRNA levels with significant change following 10 and 100 µg/ml CSE exposure (Fig. 1A). A similar change was observed in *Spdef* mRNA levels (Fig. 1B), however CSE treatment induced *Bcl-2* mRNA levels at all tested concentrations (Fig. 1C). Next, we assessed the expression kinetics of these mRNAs over 0, 3, 24, 48 and 72 h following 10 µg/ml CSE treatment. The *Muc5ac* mRNA levels were highest at 24 h post CSE treatment (Fig. 1D), and *Spdef* mRNA levels were increased within 3 h of CSE treatment (Fig. 1E). *Bcl-2* mRNA levels peaked at 48 h post CSE exposure (Fig. 1F).

Because there was a modest (~2-fold) increase in the mRNA levels, we analyzed the protein expression in murine AECs at 48 h post 10 µg/ml CSE treatment. There was increased expression of Muc5ac, Spdef and Bcl-2 in a subset of murine AECs following 10 µg/ml CSE treatment (Fig. 1G and H). CSE treatment resulted in more than 10-fold higher number of Muc5ac- and Spdef-immunopositive cells compared to non-treated controls, along with 5-fold higher Bcl-2-immunopositive murine AECs (Fig. 1H). Therefore, these results suggest that CSE exposure results in a CSE concentration- and time-dependent increase in Muc5ac, Spdef and Bcl-2 levels in murine AECs.

### Concomitant induction of MUC5AC and Bcl-2 by CSE in Human AECs

**Figure 2:**
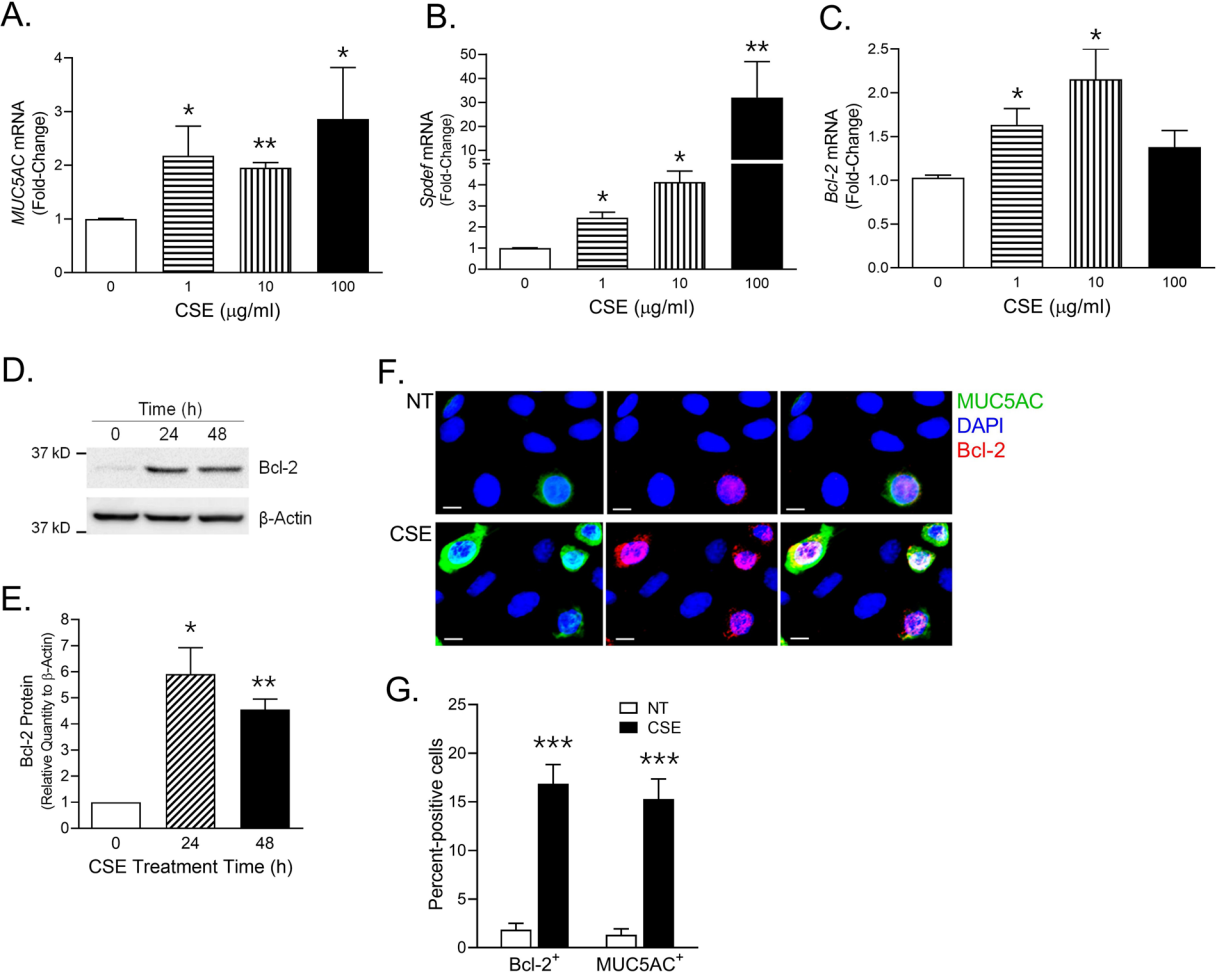
CS exposure induces MUC5AC, SPDEF, and Bcl-2 levels in human AEC monolayers. Primary human AECs grown in submerged cultured conditions were treated with 0, 1, 10, and 100 µg/ml of CSE for 24 h and the mRNA levels of *MUC5AC* (**A**) *SPDEF* (**B**) and *Bcl-2* (**C**) were analyzed by qRT-PCR. (**D**) Immunoblot analyses of Bcl-2 expression following CSE exposure. Differentiated human AECs were treated with 10 µg/ml CSE and cell lysates were analyzed at 0, 24 and 48 h post CSE exposure by western blot analysis for Bcl-2 protein levels with β-actin levels detected as the loading controls. (**E**) Relative quantities of Bcl-2 protein as determined by densitometric analysis and normalized to β-actin levels. (**F**) Representative micrographs showing MUC5AC (green) and Bcl-2 (red) immunopositivity in HAECs treated with CSE or left non-treated (NT). Human AECs were treated with CSE (10 µg/ml) for 48 h and immunostained for Bcl-2 and MUC5AC, and nuclei were stained with DAPI (Scale – 5 µ). (**G**) Quantification of human AECs immunopositive for MUC5AC and Bcl-2 following CSE exposure. The percentage of human AECs immunopositive for Bcl-2 (Bcl-2^+^) and MUC5AC (MUC5AC^+^) were calculated. Data shown as mean ± SEM (n ≥ 3); *p < 0.05; **p < 0.01; ***p < 0.001.

To characterize the CSE response in human cells, monolayers of human AECs grown in submerged cultured conditions were treated with 0, 1, 10 and 100 µg/ml of CSE for 48 h. There was a dose-dependent increase in *MUC5AC* (Fig. 2A) and *SPDEF* (Fig. 2B) mRNA levels, however, CSE treatment induced *Bcl-2* mRNA levels at 1 and 10 µg/ml CSE concentration (Fig. 2C). Bcl-2 protein levels examined by western bot analysis showed >4-fold increase following 24 and 48 h of 10 µg/ml CSE treatment (Fig. 2D and E). Specifically, following *in-situ* immunostaining, all of the MUC5AC-positive cells showed higher Bcl-2 expression at 48 h post 10 µg/ml CSE treatment (Fig. 2F). There were 5-fold higher Bcl-2^+^ and 6-fold higher MUC5AC^+^ human AECs following CSE treatment (Fig. 2G). Thus, like murine AECs, CSE exposure resulted in a concomitant increase in both MUC5AC and Bcl-2 expression in human AECs.

### CSE engages EGFR pathways to induce MUC5AC and Bcl-2 expression

**Figure 3:**
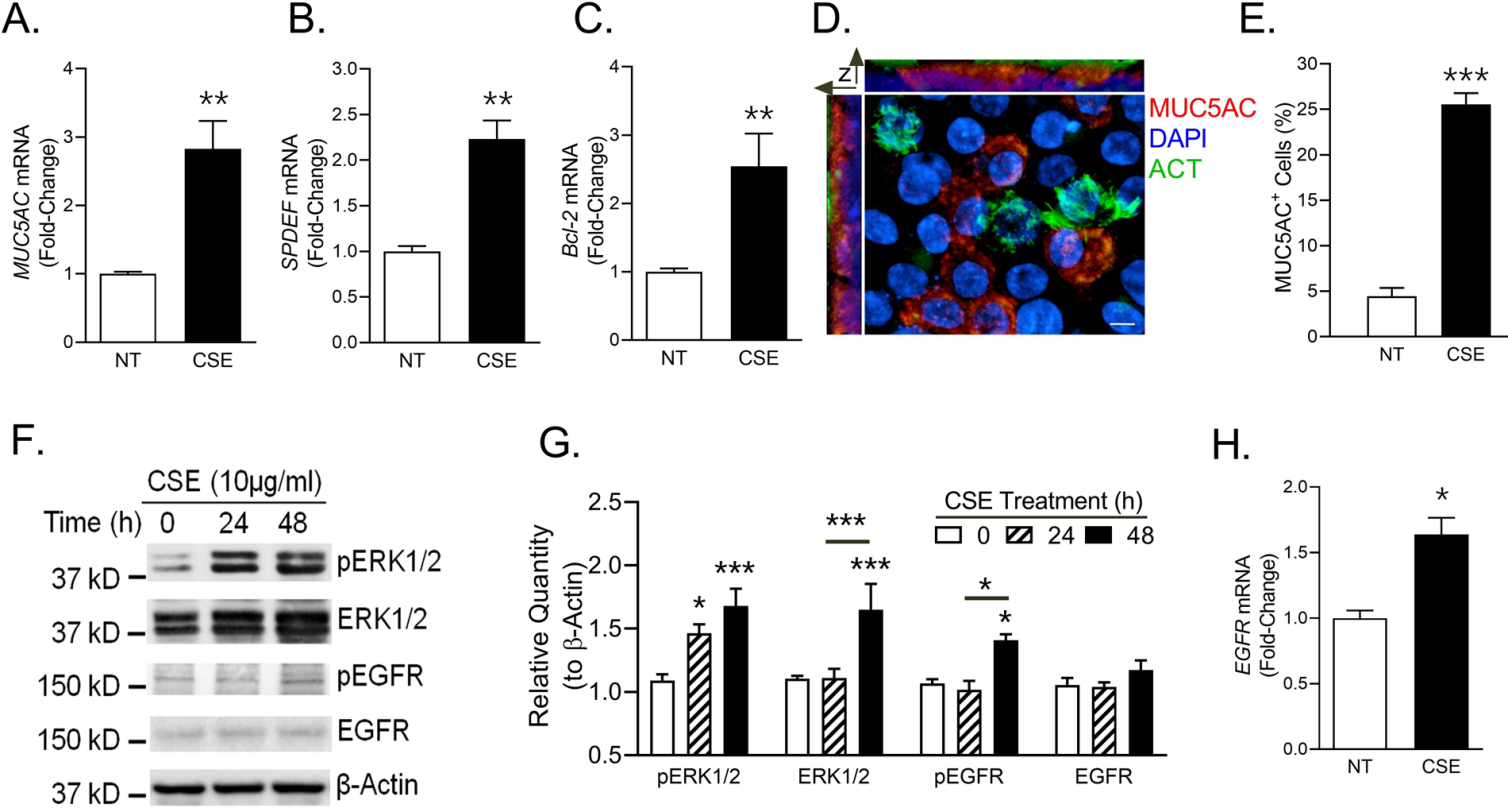
CSE engages EGFR and ERK1/2 pathways to induce mucous phenotype and Bcl-2 levels in differentiated human AECs. Primary human AECs differentiated for 3 weeks on air-liquid interface were treated with 10 µg/ml of CSE for 48 h and the mRNA levels of *MUC5AC* (**A**), *SPDEF* (**B**) and *Bcl-2* (**C**) were analyzed by qRT-PCR. (**D**) A 3-D representation of a micrograph showing cilia (green) and MUC5AC (red) immunopositivity in differentiated human AECs treated with CSE. Differentiated human AECs were treated with 10 µg/ml CSE for 48 h and were immunostained for acetylated-tubulin (ACT, in green) for cilia and MUC5AC (red), and nuclei were stained with DAPI (Scale – 5 µ). (**E**) Quantification of MUC5AC immunopositive cells following CSE exposure. Approximately 300 cells from each treatment were analyzed to calculate the percentage of MUC5AC-positive (MUC5AC^+^) cells. (**F**) Immunoblot analyses of EGFR and ERK1/2 signaling pathway following CSE exposure. Differentiated human AECs were treated with 10 µg/ml CSE and cell lysates were analyzed at 0, 24 and 48 h post CSE exposure by western blot analysis for phosphorylated ERK1/2, total ERK1/2, phosphorylated EGFR and total EGFR with β-actin levels as the loading controls. (**G**) Relative quantities of pERK1/2, ERK1/2, pEGFR and EGFR as determined by densitometric analysis where protein quantities were normalized to β-actin levels. (**H**) Fold-change in *EGFR* mRNA levels in human AECs treated with 10 µg/ml of CSE for 48 h. Data shown as mean ± SEM (n = 3); *p < 0.05; **p < 0.01; ***p < 0.001.

To mimic the *in-vivo* physiology of AECs, we next analyzed the effect of CSE on air-liquid interface differentiated human AECs. At 48 h post 10 µg/ml CSE treatment, the *MUC5AC* (Fig. 3A), *SPDEF* (Fig. 3B) and *Bcl-2* (Fig. 3C) mRNA levels were induced by CSE exposure. At protein levels too, there was increased MUC5AC expression (Fig. 3D) with a 4-fold higher number of MUC5AC^+^ cells at 48 h post 10 µg/ml CSE treatment compared to controls (Fig. 3E). We next investigated the pathways implicated in CS-induced mucus expression by analyzing EGFR (Epithelial Growth Factor Receptor) and ERK1/2 (Extracellular-signal Regulating Kinase 1/2) pathways^23,30–32^. The immunoblot analyses showed increased ERK1/2 phosphorylation at 24 and 48 h post CSE treatment whereas EGFR phosphorylation was observed only at 48 h post CSE treatment (Fig. 3F and G). *EGFR* mRNA levels were also an increased at 48 h post CSE treatment (Fig. 3H). These data thus suggest that CSE engages EGFR and ERK1/2 pathways to induce mucus and Bcl-2 expression.

### Small molecule BH3 mimetic, ABT-263, suppresses CSE-induced mucous expression by inducing apoptosis

**Figure 4:**
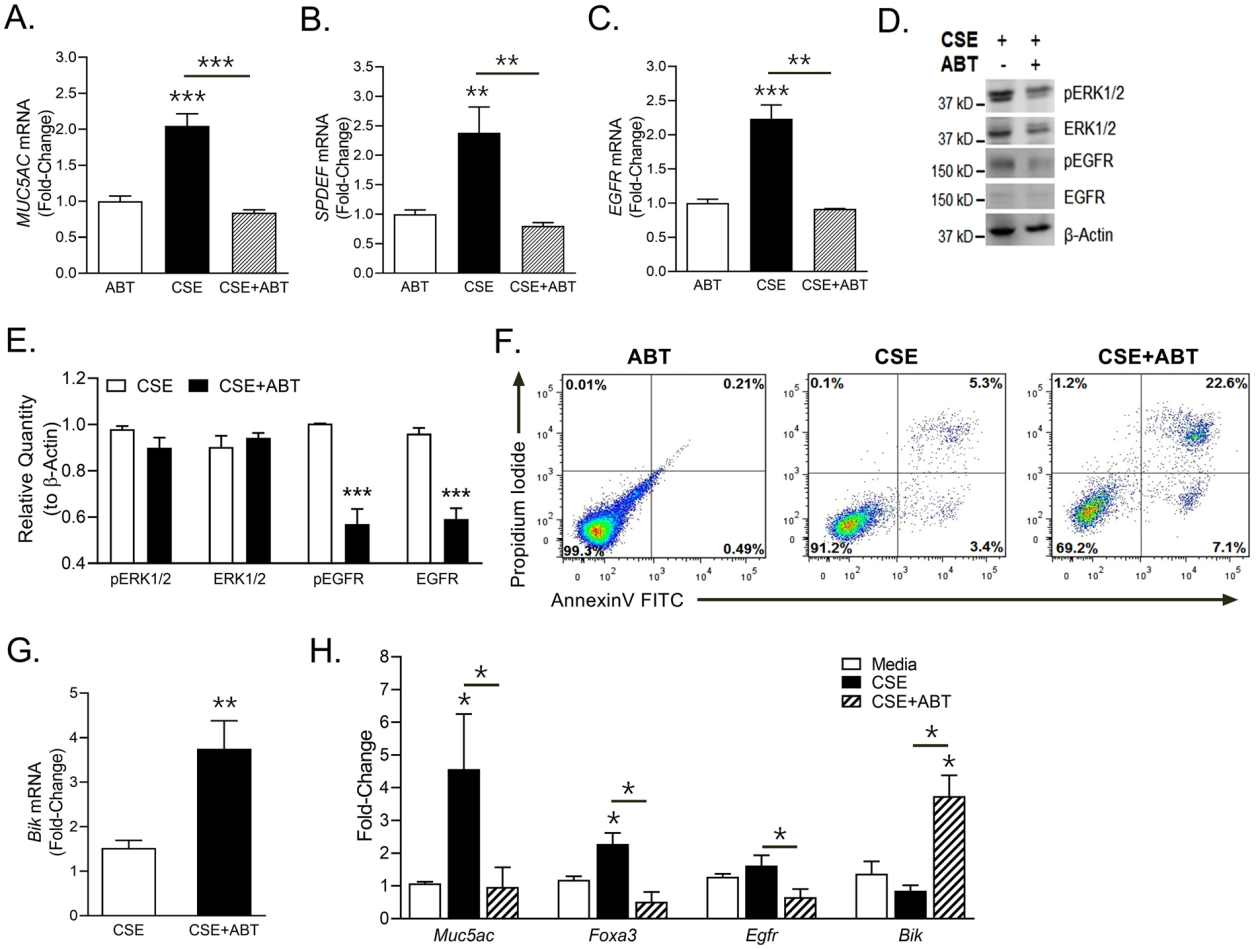
Blocking Bcl-2 with ABT-263 suppresses the CSE-induced mucous phenotype by suppressing EGFR signaling and inducing apoptosis and Bik expression. ABT-263 treatment suppresses CSE-induced *MUC5AC* (**A**), *SPDEF* (**B**) and *EGFR* (**C**) mRNA levels. Differentiated human AECs were treated with 1 µM ABT-263 for 2 h before treating with 10 µg/ml CSE and cells were harvested at 48 h post treatment. (**D**) Immunoblot analyses of pERK1/2, ERK1/2, pEGFR and EGFR following CSE exposure and ABT-263 treatment of differentiated human AECs. ALI-differentiated human AECs were treated with ABT-263 (1 µM) for 2 h before treating with 10 µg/ml CSE and cells were harvested at 48 h post treatment. (**E**) Relative quantities of pERK1/2, ERK1/2, pEGFR and EGFR as determined by densitometric analysis with protein quantities normalized to β-actin levels. (**F**) ABT-263 treatment augments apoptosis in CSE-exposed human AECs. Differentiated human AECs treated with ABT-263, CSE and CSE + ABT were harvested at 48 h post treatment, and analyzed for Annexin V-FITC and propidium iodide staining by Flow cytometry. (**G**) ABT-263 treatment induces the proapoptotic *Bik* mRNA levels that are suppressed by CSE exposure. (**H**) ABT-263 treatment of differentiated murine AECs suppresses the CSE-induced mucous secretory phenotype by modulating cell survival/death pathways. Primary murine AECs differentiated for 3 weeks on ALI were treated with ABT-263 (1 µM) and/or CSE (10 µg/ml) and the mRNA levels of *Muc5ac, FoxA3, Egfr* and *Bik* were analyzed by qRT-PCR. Data shown as mean ± SEM (n ≥ 3); *p < 0.05; **p < 0.01; ***p < 0.001.

Bcl-2 levels regulate epithelial cell survival in IL-13- and allergen-induced airway inflammation^14^ and in the present study, CSE treatment induced both MUC5AC and Bcl-2 expression. Therefore, we reasoned that blocking Bcl-2 might be helpful in restricting the CS-induced mucous response. We had tested various chemical and molecular approaches to inhibit Bcl-2 and found that ABT-263, a small molecule BH3-domain mimetic compound efficiently blocks Bcl-2 activity with fewer off-target effects^14^. Therefore, we tested the effect of ABT-263 on air-liquid interface differentiated human AECs. Pretreatment with ABT-263 (1uM) significantly suppressed the CSE-induced *MUC5AC* (Fig. 4A), *SPDEF* (Fig. 4B) and *EGFR* (Fig. 4C) mRNA as observed in CSE + ABT treatment group with no discernible effect in ABT-only treated cells. ABT-263 pretreatment suppressed the CSE-induced EGFR pathway by 2-fold in CSE + ABT condition compared to CSE only group and there were no changes in ERK1/2 pathway (Fig. 4D and E).

Because ABT-263 is a known apoptotic inducer, therefore, we examined the extent of apoptosis in our experimental setting by flow cytometric analysis of the Annexin V and propidium iodide stained cells. There were 4-fold higher apoptotic cells in human AECs pretreated with ABT-263 after CSE exposure (CSE + ABT) compared to CSE- or ABT-alone treated controls (Fig. 4F). Our previous studies have shown that Bik is a key proapoptotic protein in AECs that induces cell death^14,16^ and therefore, we analyzed the effect of ABT-263 on Bik levels. Compared to CSE treated cells, the *Bik* mRNA levels were upregulated in CSE + ABT treated cells (Fig. 4G). Similarly, in differentiated murine AECs (Fig. 4H), ABT-263 significantly suppressed the CSE-induced expression of *Muc5ac, Egfr* and *FoxA3*, another mucous-regulating transcription factor^33^. In addition, there were higher *Bik* mRNA levels in CSE + ABT treated murine AECs (Fig. 4H).

### ABT-263 completely blocks CS-induced mucous in 3-D human airway tissue

**Figure 5:**
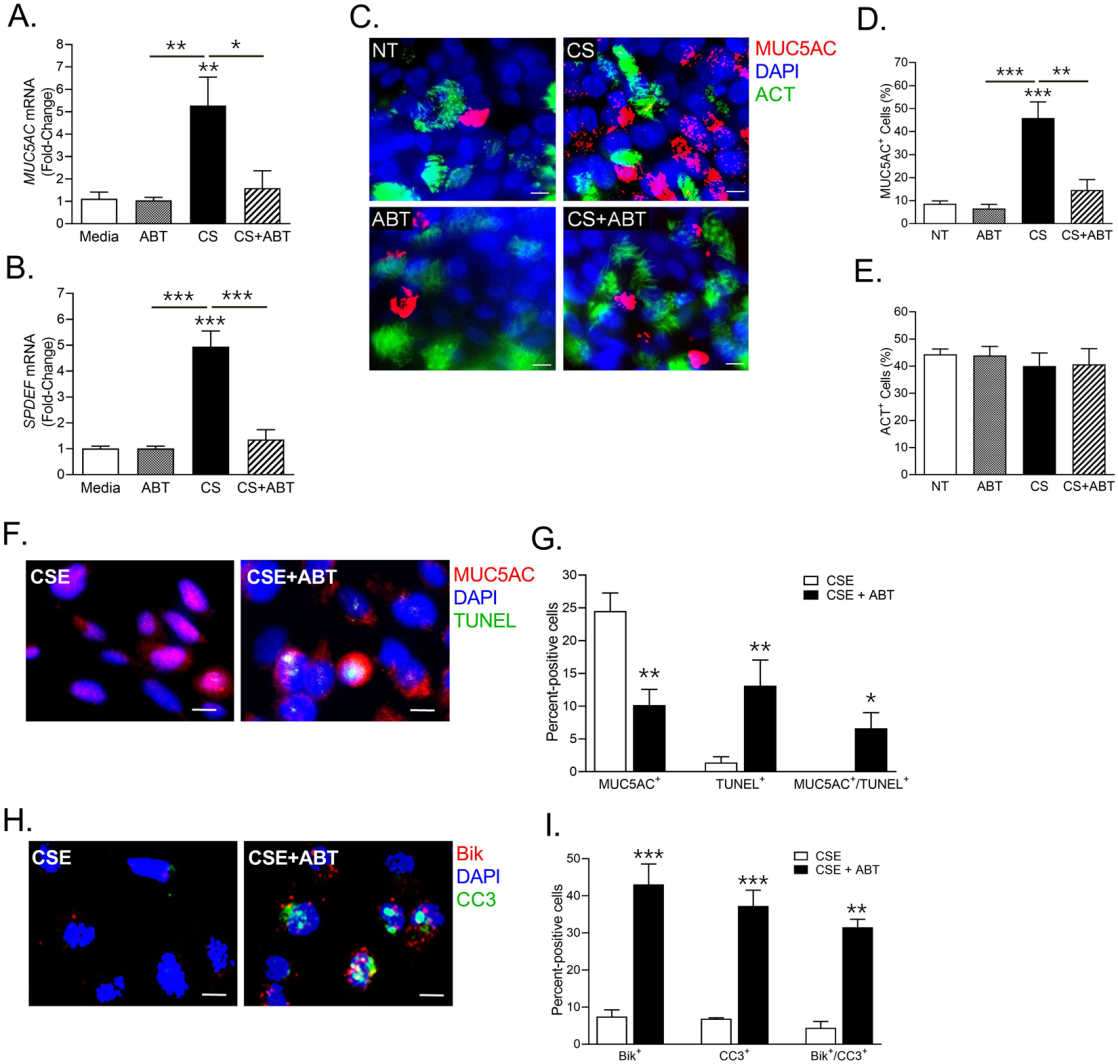
BH3 mimetic ABT-263 blocks the CS-induced mucous phenotype in 3-D human airway tissue model via inducing apoptosis and Bik expression. 3-D tissue cultures of human airways were exposed to CS using a SCIREQ smoke machine (Montreal, QC, Canada) for 3 consecutive days and one group was treated with 1 µM ABT-263 2 h before exposures. ABT-263 treatment inhibited the CS-induced *MUC5AC* (**A**) and *SPDEF* (**B**) mRNA expression. (**C**) Representative micrographs showing cilia (green) and MUC5AC (red) immunopositivity in 3-D airway tissue culture treated with CS and/or ABT-263 compared to non-treated (NT) ones. 3-D airway tissues were immunostained for acetylated-tubulin (ACT, in green) for cilia and MUC5AC (red), and nuclei were stained with DAPI (Scale – 5 µ). Quantification of MUC5AC + mucous cells (**D**) and ACT + ciliated cells (**E**) where more than 300 cells from each treatment were analyzed. (**F**) ABT-263 treatment results in increased TUNEL-positivity in CSE-exposed human AECs. Human AECs treated with ABT-263 (1 µM) and/or CSE (10 µg/ml) were stained for TUNEL (green) and MUC5AC (red). (**G**) Quantification of human AECs immunopositive for MUC5AC (MUC5AC^+^) or TUNEL (TUNEL^+^) or both (MUC5AC^+^/TUNEL^+^) following ABT-263 and CSE treatment. (**H**) ABT-263 increases Bik expression and caspase 3 activation in CSE-exposed AECs. Human AECs were immunostained for Bik (red) and cleaved caspase 3 (CC3, shown in green). (**I**) Quantification of human AECs immunopositive for Bik (Bik^+^) or CC3 (CC3^+^) or both (Bik^+^/CC3^+^) following ABT-263 and CSE treatment. Data shown as mean ± SEM (n ≥ 3); *p < 0.05; **p < 0.01; ***p < 0.001.

In order to assess the efficacy of ABT-263 in blocking CS-induced mucous, we analyzed the 3-D EpiAirway tissue culture model (MatTek Corp, Ashland, MA). The 3-D human epithelial airway tissues were exposed to mainstream CS using a SCIREQ smoke machine (Montreal, QC, Canada) for three consecutive days and a group of tissues were treated with 1 µM ABT-263 treatment 2 h before exposures. Smoke exposures caused around 5-fold increase in both *MUC5AC* (Fig. 5A) and *SPDEF* (Fig. 5B) mRNA expression which was completely blocked by ABT-263 pretreatment. There was a significant increase in MUC5AC^+^ cell population (40% cells) following smoke exposure (Fig. 5C and D) that was suppressed in ABT-263 pretreated tissues (15% cells) with no discernible changes in ciliated cell population (Fig. 5C and E). Thus, blocking Bcl-2 by a small molecule BH3 mimetic, ABT-263 suppresses the smoke-induced mucous phenotype without affecting the ciliated epithelial cells.

### ABT-263 induces Bik expression and apoptosis in CSE-induced mucous cells

To determine whether CSE-induced mucous cells were undergoing apoptosis, human AECs pretreated with ABT-263 and exposed to CSE for 48 h were analyzed by TUNEL (Terminal deoxynucleotidyl transferase dUTP nick end labeling) assay and were co-stained for MUC5AC (Fig. 5F). There were >10-fold higher number of TUNEL-positive cells in CSE + ABT treated cells with majority of MUC5AC^+^ cells showing TUNEL positivity as represented by MUC5AC^+^TUNEL^+^ cell counts (Fig. 5G). Next, we analyzed whether Bik expression is associated with the apoptotic AECs by co-staining the CSE + ABT and CSE-treated cells for Bik and activated or cleaved caspase 3 (CC3), a late-stage apoptosis indicator (Fig. 5H). There were higher number of cells expressing Bik (Bik^+^) in CSE + ABT treatment condition with most of Bik^+^ cells were undergoing apoptosis as detected by the CC3 immunostaining and are represented as Bik^+^CC3^+^ cell counts (Fig. 5I). These data suggest that ABT-263 treatment of CSE exposed cells suppresses mucous expression by upregulating Bik levels and augmenting apoptosis.

## Discussion

In this study, we report that CS exposure induces MUC5AC mucin and SPDEF, the mucous master regulator with a concomitant induction in Bcl-2 levels in both human and murine airway epithelial cells. The treatment with a small molecule BH3 mimetic compound, ABT-263, attenuated the CS-induced mucus expression in both human and murine cells. Furthermore, we observed that ABT-263 treatment attenuated the CS-engaged EGFR signaling to help upregulate apoptosis and the proapoptotic Bik levels in order to suppress the CS-induced mucus expression.

CS exposure has been shown to drive chronic mucus production^6,11,28,34–36^ and alters the cell fate by affecting the cell proliferation and the cell death pathways^13,16,26,27^. During the lung development, a network of transcription factors including thyroid transcription factor-1 (*TTF1*), forkhead box protein A2 (FOXA2) and A3 (FOXA3), and SPDEF, drive the fate of respiratory airway epithelial cells^29,37,38^. This cybernetic transcriptional network regulates the proliferation, differentiation and function of airway epithelial cells including mucous, ciliary and basal cells. Among these transcription factors, SPDEF is the important regulator of the growth and differentiation of mucous cells and mucus production^29^ and its levels are upregulated in airway mucous cells of patients with chronic airway diseases^29^ and in animal models of allergen exposure^17^. In our studies using CSE exposure model, both murine and human airway epithelial cells showed a dose-dependent response in SPDEF levels, one of the first reports showing the direct effect of CS on this mucous master regulator. It is notable that human airway epithelial cells were more sensitive to CSE exposure than murine counterparts because 1 µg/ml CSE, the lowest dose tested, significantly induced both MUC5AC and SPDEF levels in humans with no discernable changes in murine airway epithelial cells. Moreover, even at the higher CSE dose there was only modest increase in murine *Muc5ac* and *Spdef* expression compared to human AECs. This could be due to the inherent differences between human and murine airway epithelial physiology and the mucous regulatory network as discussed in detail elsewhere^8,39^. In our previous studies, we observed that murine AECs inherently show moderate mucous response due to a mucous-limiting p53 genotype that negatively affects *Bcl-2* mRNA half-life and SPDEF transcriptional activity^15^. Nonetheless, Bcl-2 seems to be important for mucous expression in both human and murine airway epithelial cells because ABT-263 treatment blocked the mucous expression in cells of both origin. With respect to our studies, the absence of SPDEF in a mice model was shown to attenuate the allergen-induced mucous cell development and mucus production^29,40^. Similarly, knockdown of SPDEF with small interfering RNA (siRNA) in the human bronchial epithelial cell line was found to significantly reduce the expression of IL-13-induced MUC5AC expression^41^. Recently, in order to achieve a long-lasting mucus inhibition Song *et al*. have successfully used epigenetic silencing of SPDEF to downregulating mucous expression in human lung epithelial cells^42^. These observations together with our data suggest that SPDEF could be a potential therapeutic target for regulating CS-induced mucus hypersecretion and future studies will help determine the feasibility of this proposition.

Cigarette smoke components engage several signaling events that lead to increased differentiation and proliferation of mucous cells leading to chronic mucus production^43,44^ and here, we observed that EGFR might be involved in CSE-induced mucin expression. In addition, CS induced inflammatory mediators secreted from airway epithelial cells might be providing a feed-forward to the EGFR-mediated mucin expression in an autocrine or paracrine manner because various EGFR ligands are present in AECs in an inactive form^45^. Furthermore, CS stimulates reactive oxygen species or ROS production that induce TGFα and activation of TNFα-converting enzyme (TACE)-mediated mucin expression^30,46,47^. Similarly, dual oxidase-1 (DUOX-1) is also expressed by airway epithelium that could activate TACE to engage EGFR-mediated mucin expression^48^. More importantly, EGFR pathways has been identified as key proinflammatory mediators that aide in epithelial and mucous cell hyperplasia^23,49–53^ and are associated with the survival of airway epithelial cells in response to a viral challenge^49,54^. We observed an increased phosphorylation of EGFR following CSE exposure that could be implicated in the subsequent induction of both Bcl-2 and MUC5AC. These findings are consistent with other studies, which report TGFα-induced activation of EGFR mediates Bcl-2 induction^52,53^. Moreover, the external stimuli including exposure to CS, LPS, ozone, or allergen that upregulate Bcl-2 expression, also activate EGFR in airway epithelial cells^47,54^. Recently, we reported that when airway epithelial cells were pre-incubated with the specific EGFR and ERK1/2 inhibitors there was a significant suppression of the allergic inflammation induced expression of MUC5AC and Bcl-2^14^. However, in the present study we failed to see any significant changes in ERK1/2 signaling following ABT treatment although CSE exposure caused increased ERK1/2 phosphorylation. There are other ERK-independent pathways engaged by CS and CS-induced ROS to help induce MUC5AC including JNK and AP-1 signal transducers^31^. Further studies are required to delineate the CSE-engaged EGFR signaling mediator(s) that result in mucus expression specifically the one blocked by BH-3 mimetic compound treatment.

Collectively, the present study suggests that Bcl-2 and MUC5AC may be regulated by identical or overlapping pathways implicating EGFR as one of the upstream mediators. Besides epithelial cell proliferation and survival signaling, EGFR mediates MUC5AC synthesis following exposure to CS-, LPS-, or microbial infection^30,55^. Therefore, EGFR activation might be one the primary mechanism responsible for the induction of mucous cell differentiation^52,53^ following CS exposure to help sustain hyperplastic mucous cells. However, therapeutic approaches targeting EGFR receptors face considerable safety and efficacy challenges in clinical trials because COPD subjects poorly tolerated EGFR inhibitor, BIBW 2948, and more so, there was no discernible reduction in mucin production^56^. Therefore, downstream mediators of EGFR signaling may offer a more effective target for developing novel mucolytic therapeutics.

One such target could be Bcl-2, which is critical for airway inflammation induced mucous cell hyperplasia^14,20,21^. Bcl-2 is a founding member of a family of proteins that maintain cellular homeostasis by regulating programmed cell death pathways like apoptosis and autophagy^18,57^. Bcl-2 protects cells against a wide range of cell death stimuli by stabilizing the mitochondrial and endoplasmic reticulum (ER) membrane, and preventing permeabilization and release of death mediators^58,59^. Bcl-2 is present in the outer mitochondrial and ER membranes, and inactivates BH3-domain consisting pro-apoptotic members of the Bcl-2 family^58,59^. Here, using a BH3 mimetic compound, ABT-263, which blocks Bcl-2 activity by competing for the BH-3 binding domain with proapoptotic proteins resulting in increased apoptosis; we were able to suppress the CSE-induced mucus expression. Moreover, among the CSE-exposed differentiated airway epithelial cells, there was increased apoptosis in ABT-263-treated ones compared to non-treated controls accompanied by increased Bik levels and reduced EGFR phosphorylation. Bik is the known proapoptotic protein in airway epithelium because Bik-deficient mice fail to resolve allergen-induced mucous expression^60^. In airway epithelium of patients with chronic airway diseases, there is significant reduction in *Bik* mRNA compared to controls^16^. Therefore, ratio of Bik and Bcl-2 levels could be crucial in determining the epithelial and mucous cell fate because blocking Bcl-2 with ABT-263 results in Bik-mediated cell death.

The present study posits several limitations that merit further discussion. Firstly, the CS extract is primarily consisted of the solution phase extract and do not replicate the actual gaseous and particulate exposure conditions. In addition, there are wide variations in the CS extraction methods. In our studies, we employed both organic and aqueous extraction method to prepare the CS extract and observed that 10 µg/ml concentration was optimal to observe mucous response without altering cell growth. This concentration when extrapolated was equivalent to a 100 µg/mm^3^ of actual CS exposure; the levels comparable to those active smokers get exposed to. Secondly, in this study, we have only observed the effect of ABT-263 in cell culture model and to establish any clinical relevance of our findings, the preclinical animal model testing is required. However, like humans where CS-mediated effects take decades, the mouse models also require longer duration of CS exposure to observe any phenotypic changes in airway responses. From our previous time-course *in-vivo* studies in a mouse model of CS exposure^20^, we had observed that only after 16 weeks of exposure there was an observable mucous response. These longer exposure conditions might need a continuous sustained release of ABT-263 to avoid daily treatment and added irritation to animals. We are currently developing a sustained-release regimen of ABT-263 to help conduct these studies. Nonetheless, our *in-vitro* data using 3-D human airway tissues and differentiated AECs do provide a strong support for the utility of ABT-263 in blocking CS-induced mucous conditions. With the current success of ABT-263 in the treatment of human cancer and other chronic diseases^61,62^, it will be safer to presume that these BH3 mimetics might find the utility in regulating mucous pathologies associated with chronic airway diseases.

## Methods

### Cell Culture

Murine AECs were isolated from mouse trachea and cultured on plastic plate or Transwell membranes (Corning, New York, NY) as described previously^63^. All experimental procedures were carried out in accordance with FIU institutional guidelines and regulations. Briefly, tracheas from C57Bl/6 (Jackson Laboratory) mice were excised, connective tissue was cleared, and tracheas were cut open lengthwise. Cleaned tracheas were incubated in Pronase solution (DMEM, 1.4 mg/mL Pronase, and 0.1 mg/mL DNase) overnight at 4 °C to dissociate AECs from BL. Enzymatic activity was stopped with 10% FBS (Invitrogen, Carlsbad, CA), and cells were collected by gently rocking trachea in DMEM/Ham H12 media (Invitrogen), followed by centrifugation at 400 g for 10 minutes at 4 °C. Cell clumps were dissociated by using 5 mL of declumping solution (DMEM and 2 mmol/L EDTA) and plated at 100,000 cells/well on collagen-coated plates. Primary human AECs were kindly provided by Dr. Scott Randell at the Marsico Lung Institute/Cystic Fibrosis Research Center at the University of North Carolina, Chapel Hill, USA. Lung tissues were procured under protocol #03-1396 approved by the University of North Carolina at Chapel Hill Biomedical Institutional Review Board; informed consents were obtained from all subjects and AECs were procured as previously described^64^. The AECs were maintained in bronchial epithelial growth medium (BEGM, Lonza, Walkersville, MD). For air-liquid interface culture, AECs were seeded on Transwell membranes and differentiated for 14 days. Following treatments, the membrane quarters were used for RNA and protein isolation and were fixed in 4% paraformaldehyde for immunostaining. All the methods were performed in accordance with the institutional guidelines and regulations approved by FIU.

### Preparation of Cigarette Smoke Extract and Treatment

The cigarette smoke particulate matter from research-reference filtered cigarettes (3R4F; University of Kentucky, KY, USA) collected on Cambridge glass fiber filters were kindly provided by Dr. P. Kuehl (Lovelace Biomedical, Albuquerque, NM). The CS extract amount obtained was determined by weight increase of the filter. CSE was prepared by dissolving the collected smoke particulates in BEGM media and dimethyl sulfoxide (DMSO) to yield a 200 µg/ml (w/v) solution and aliquots were stored at −80 °C.

### Cigarette Smoke Exposure

The 3-D EpiAirway tissue cultures (MatTeck Corp, Ashland, MA) were exposed to mainstream CS using a SCIREQ smoke machine (Montreal, QC, Canada). Four 3R4F research-reference filtered cigarettes (University of Kentucky) were smoked with a puff volume of 35 ml per 2 sec for every minute and blown over tissue culture rate of 5 ml/min in basic conformity with ISO 3308 (International Organization for Standardization, 2012a). Total 32 puffs were recorded for a duration of approximately 35 minutes of exposure per day. Smoke exposures were performed for 3 consecutive days with1 µM ABT-263 treatment 2 h before exposures and tissues were analyzed at 48 h post last exposure.

### Immunostaining and Fluorescent Imaging Analysis

The murine and human AECs grown on Labtek-II slides (ThermoFisher Inc.) were fixed in 4% paraformaldehyde and washed in 0.05% v Brij-35 in PBS (pH 7.4) and immunostaining was performed as described previously^14^. Briefly, the cells were blocked using a solution containing 3% BSA, 1% Gelatin and 1% normal donkey serum with 0.1% Triton X-100 and 0.1% Saponin and were stained with antibodies to MUC5AC (Millipore Inc., Burlington, MA), Spdef (Santa Cruz Biotech, Dallas, TX), Bcl-2 (Santa Cruz Biotech, Dallas, TX), Bik (Abcam, Cambridge, MA) and cleaved caspase 3 (Cell Signaling Tech., Danvers, MA) or isotype controls. The immunolabelled cells were detected using respective secondary antibodies conjugated fluorescent dyes (Jackson ImmunoResearch Lab Inc., West Grove, PA) and mounted with 4′,6-diamidino-2-phenylindole (DAPI) containing Fluormount-G^TM^ (SouthernBiotech, Birmingham, AL) for nuclear staining. Immunofluorescent images were captured using BZX700 Microscopy system (Keyence Corp., Japan) and analyzed using NIH Image J software.

### Flowcytometric Quantification of Apoptosis

Apoptotic cells were quantified by fixing cells in 2% paraformaldehyde and staining with Annexin V-FITC conjugate (BD Biosciences Inc., San Jose, CA) and propidium iodide (Sigma-Aldrich Inc., St. Louis, MO) for 30 min at 4 °C. Stained cells were washed and resuspended in 0.2% BSA/PBS and nearly 10,000 cells per sample were analyzed using BD FACS Canto® Flow Cytometer (BD Biosciences Inc., San Jose, CA) and the data was analyzed using FlowJo analysis software (Tree Star Inc., Ashland, OR).

### Quantitative Real-Time RT-PCR

Total RNA was isolated from the snap-frozen cells using RNAeasy kit (Qiagen, Germantown, MD) as per manufacturer’s instruction. RNA concentration was determined using the Synergy HTX Multi-Mode reader (BioTek, Winooski, VT) and cDNA were synthesized using iScript advanced cDNA kit (BioRad, Hercules, CA). The primer/probe sets for MUC5AC, SPDEF, FOXA3, and Bcl-2 were obtained either from BioRad (Hercules, CA) or Qiagen (Germantown, MD) and cDNA amplified by q-PCR using the iTaq SYBR-green Master Mix (BioRad, Hercules, CA) in the Agilent Stratagene Mx3000P Real-Time PCR System (Thermo Fisher Scientific, Waltham, MA). Relative quantities were calculated by normalizing averaged C_T_ values to GAPDH or β-Actin to obtain ΔC_T_, and the fold-change (ΔΔC_T_) over the controls were determined as described previously^15^.

### Western Blot Analysis

Cell extracts were prepared using RIPA buffer (20 mM Tris, pH 7.4, 137 mM NaCl, 1% NP-40, 0.25% Deoxycholate, 0.1% SDS, 1 mM EDTA and 1% protease inhibitor cocktail). Protein concentration was determined by BCA kit (Pierce; Rockford, IL) and 50 µg protein was analyzed by western blotting as described previously^15^. Antibodies to Bcl-2, EGFR, p-EGFR, ERK1/2 and p-ERK1/2 were all from Cell signaling technologies (Danvers, MA) and β-actin antibody was from Sigma co. (St. Louis, MO). Proteins were detected using ECL and visualized by chemiluminescence (Perkin Elmer, Waltham, MA) using the BioRad Chemidoc Imaging system (Hercules, CA). The full-length immunoblots are available online as supplemental information.

### *In-Situ* Apoptosis Detection by TUNEL

Paraformaldehyde-fixed cells were washed in 0.05% v Brij-35 in PBS (pH 7.4) and processed for TUNEL labelling as per manufacturer’s instruction using TACS•XL® *In-Situ* Apoptosis Detection Kit (Trevigen, Gaithersburg, MD). The TdT-labelled cells were detected using fluorescently-conjugated secondary antibodies (Jackson ImmunoResearch Lab Inc., West Grove, PA). The cells were counterstained with 4′,6-diamidino-2-phenylindole (DAPI) containing Fluormount-G (SouthernBiotech, Birmingham, AL) to visualize nuclei. Images were captured with BZX700 Microscopy system (Keyence Corp., Japan) and analyzed by NIH Image J software.

### Statistical Analysis

Grouped results were expressed as means ± SEM. Data were analyzed using GraphPad Prism Software (GraphPad Software, Inc., San Diego, CA). Grouped results were analyzed using two-way analysis of variance. When significant main effects were detected (*P* < 0.05), Fishers least significant difference test was used to determine differences between groups.

## Electronic supplementary material

Supplementary Data
