# Supplementary material for: A Small Molecule BH3-mimetic Suppresses Cigarette Smoke-Induced Mucous Expression in Airway Epithelial Cells
Source: Sci Rep. 2018 Sep 14;8:13796. doi: 10.1038/s41598-018-32114-w (PMC6138652; doi:10.1038/s41598-018-32114-w)
Supplement: Supplementary file 1 — Supplementary Data [file 41598_2018_32114_MOESM1_ESM.pdf]

Online Supplemental File

A Small Molecule BH3-mimetic Suppresses Cigarette Smoke-Induced Mucous Expression in Airway Epithelial Cells

Shah S. Hussain [1], Shebin George [1], Shashi Singh [2], Rahul Jayant [1], Chien-An Hu [3], Mohan Sopori [2], and Hitendra S. Chand [1,*]

[1] Department of Immunology, Herbert Wertheim College of Medicine, Florida International University, Miami, FL - 33199, USA. [2] Lovelace Respiratory Research Institute, Albuquerque, NM - 87108, USA, [3] Department of Biochemistry and Molecular Biology, University of New Mexico, NM - 87131, USA.

***Corresponding Author:**

Hitendra S. Chand, PhD
Department of Immunology,
Herbert Wertheim College of Medicine,
Florida International University,
Miami, FL 33199
Tel: (305) 348-1472
E-mail: hchand@fiu.edu

Funding: NIH R21 AI117560 (H.S.C) and American Lung Association RG306208 (H.S.C.)

Fig. 2D

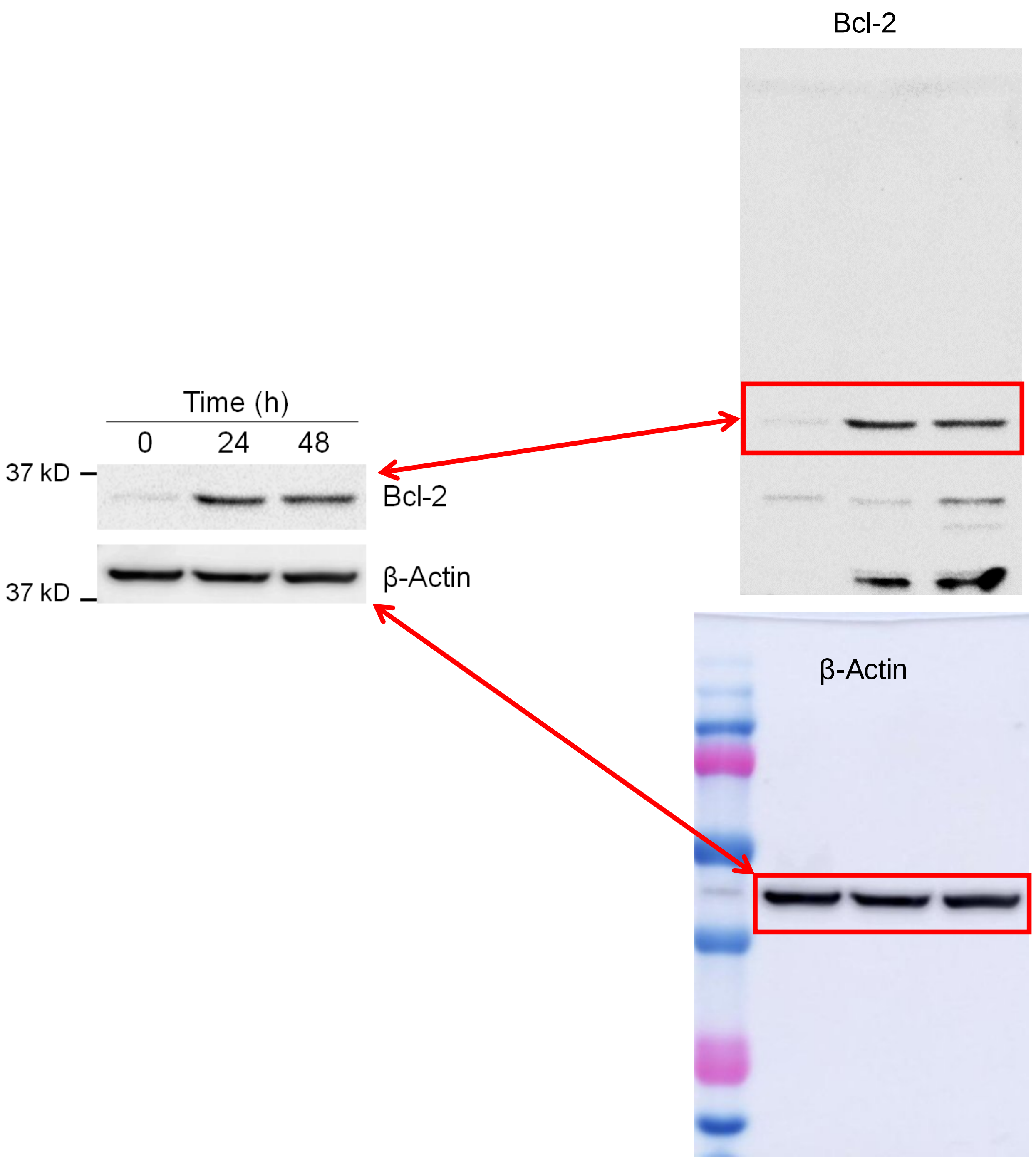

Fig. 3F

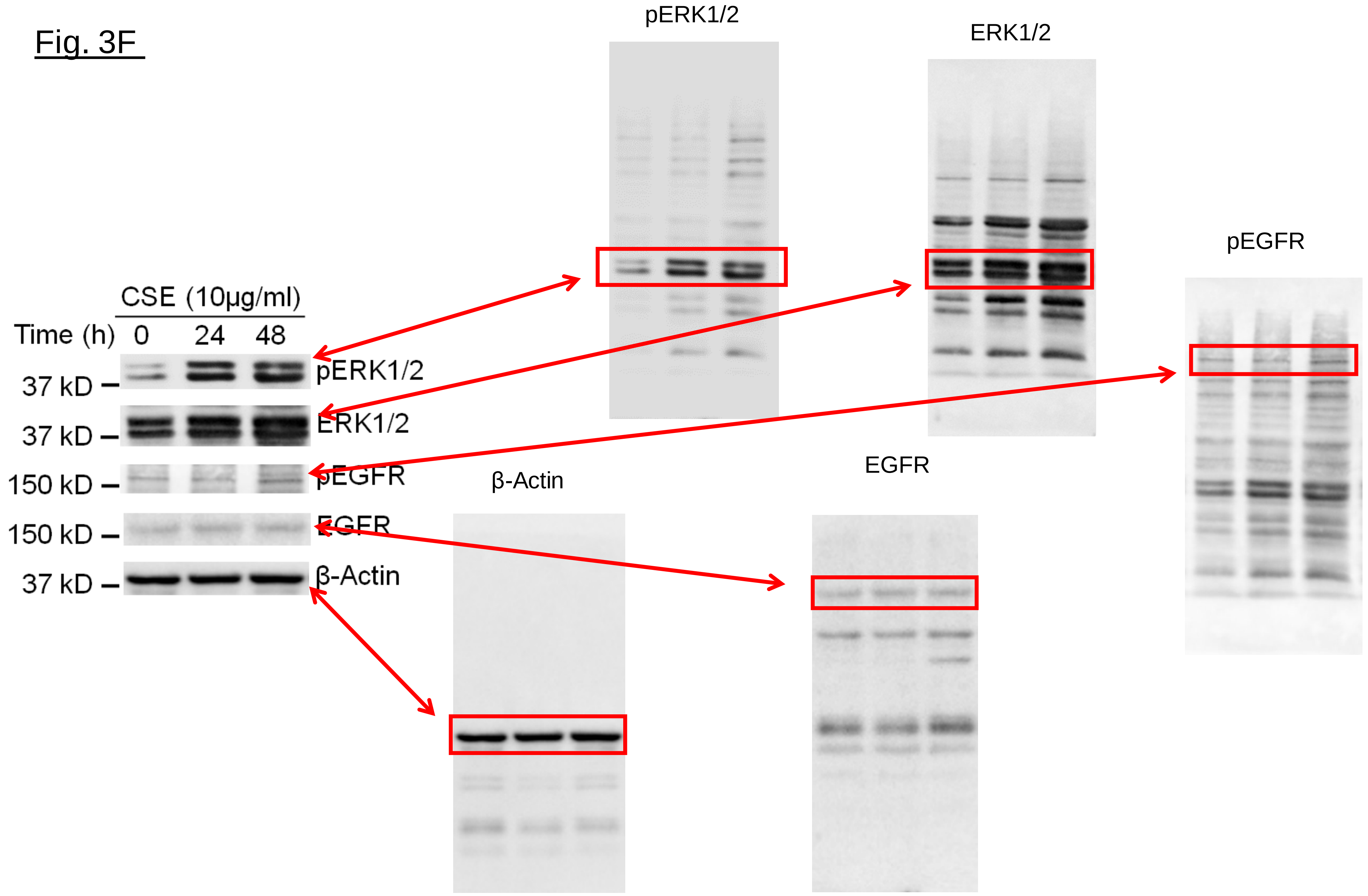

CSE (10μg/ml)
Time (h) 0 24 48
37 kD
pERK1/2
37 kD
ERK1/2
150 kD
pEGFR
150 kD
EGFR
37 kD
β-Actin
pERK1/2
ERK1/2
pEGFR
β-Actin
EGFR

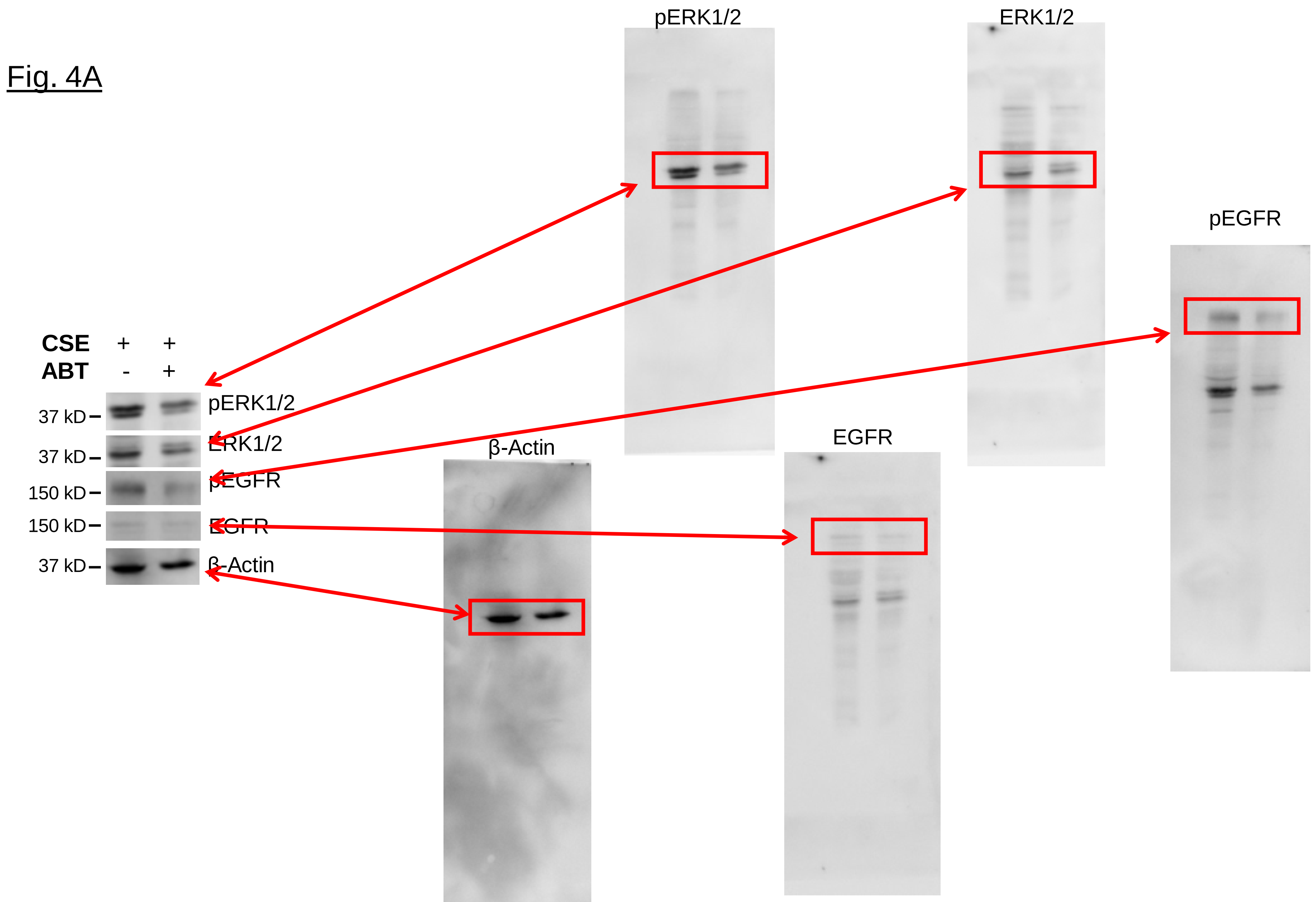
Fig. 4A
CSE + +
ABT - +
37 kD
37 kD
150 kD
150 kD
37 kD
pERK1/2
ERK1/2
pEGFR
EGFR
β-Actin
pERK1/2
ERK1/2
pEGFR
EGFR
β-Actin
